# Symptomatic Congenital Kyphoscoliosis Due to Concomitant Wedged Vertebra and Sotos Syndrome: A Case Report

**DOI:** 10.7759/cureus.71743

**Published:** 2024-10-17

**Authors:** Shimei Tanida

**Affiliations:** 1 Spine Surgery, Shiga General Hospital, Shiga, JPN

**Keywords:** congenital kyphoscoliosis, paraplegia, pediculectomy, sotos syndrome, syndromic scoliosis

## Abstract

There are few reports of syndromic scoliosis accompanied by a congenital vertebral anomaly. We report a case of Sotos syndrome with a concomitant congenital wedged vertebra whose kyphoscoliosis progressed rapidly and presented with myelopathy during the growth-spurt period. A 12-year-old male suffering from Sotos syndrome with T10-wedged vertebra presented with paraparesis and urinary dysfunction. Magnetic resonance imaging showed that the spinal cord was deviated to the concave side and was compressed from the right pedicle and posterior wall of the vertebral body at T10 level. He underwent direct decompression surgery including posterior right T10 pediculectomy and corrective fixation from T5 to L1. After the surgery, his gait disturbance improved, and he no longer had urinary incontinence. His kyphoscoliosis also improved.

Two congenital diseases, Sotos syndrome and congenital vertebral anomaly, such as in the present case, require careful follow-up or early surgical intervention, especially during the growth-spurt period, to avoid missing the good timing for surgery because of the possibility of rapid deterioration of kyphoscoliosis and subsequent spinal cord disorders.

## Introduction

Sotos syndrome is a congenital anomaly syndrome that presents with large macrocephaly, overgrowth, accelerated bone age, mental retardation, delayed motor development caused by muscle hypotonia, seizures, heart disease, urinary tract abnormalities, and kyphoscoliosis due to haploinsufficiency of the NSD1 gene, which is located in the 5q35 region of the long arm of chromosome 5. The incidence of this disease is estimated to be about one in 10,000 to 15,000 births [1,2]. The frequency of scoliosis in Sotos syndrome is high, and it is important to carefully monitor patients during their growth spurt. Due to the hyperextension of the joints and hypotonia characteristic of this syndrome, the scoliosis curve tends to be large.

Congenital scoliosis, on the other hand, is present at a rate of 0.5-1/1000 births and accounts for 10% of all scoliosis cases [3,4]. Embryologically, scoliosis is an abnormality of somitogenesis and is often complicated by malformations of the urinary tract, musculoskeletal system, heart, and ribs. Congenital scoliosis is classified into four types: Type 1 - formation failure; Type 2 - segmentation failure; Type 3 - mixed type; and Type 4 - unclassifiable [3]. Of these, deformities including type 1 may present with severe rigid angular deformity, which may be neurologically aggravated in 18% of cases due to spinal cord compression [4]. Paraplegia is especially likely to occur around puberty, at an average age of 13 years. Among the formation failures, posterolateral quadrant hemivertebra, posterior hemivertebra, wedge vertebra, and butterfly vertebra are the most likely to develop, in that order. It is reported that wedged vertebrae are concentrated in the thoracic and thoracolumbar vertebrae, and their rate of progression is variable but relatively slow, 1-2° per year [3].

We report a case of Sotos syndrome with a concomitant T10-wedged vertebra that developed paraparesis and urinary dysfunction during follow-up and underwent direct decompression surgery including posterior pediculectomy and corrective fixation.

## Case presentation

**Figure 1:**
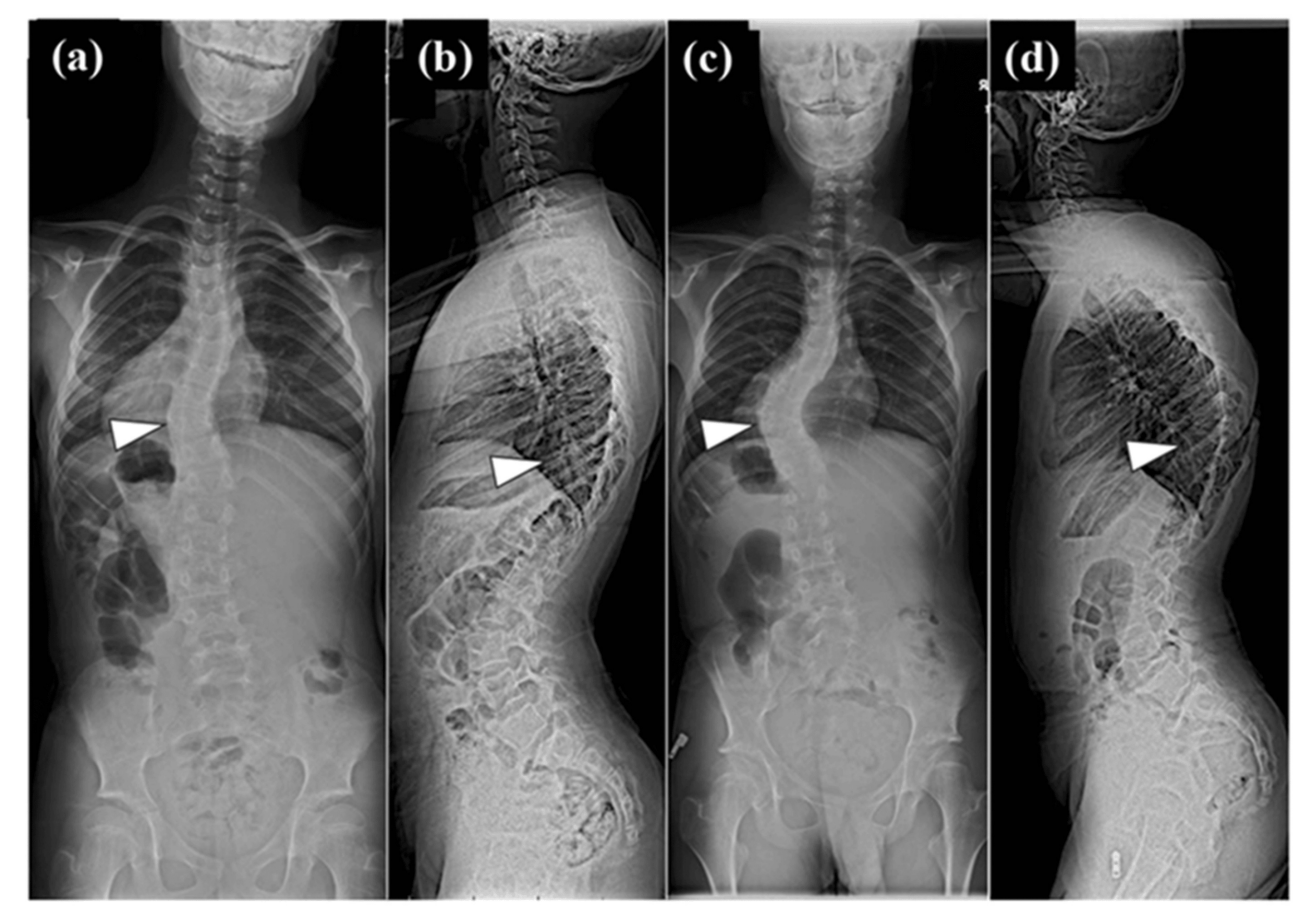
Coronal and sagittal standing full-spine radiograph taken 1.5 years before and at the time of surgery. Coronal standing full-spine radiograph taken 1.5 years before surgery (a) and at the time of surgery (b) showed the Cobb angle of the scoliotic major curve from T9 to T11 was 35 degrees and 60 degrees, respectively. Sagittal standing full-spine radiograph taken 1.5 years before surgery (c) and at the time of surgery (d) showed the Cobb angle of the local kyphosis from T8 to T11 was 35 degrees and 60 degrees, respectively. The white triangle indicated the T10 vertebra in each image.

**Figure 2:**
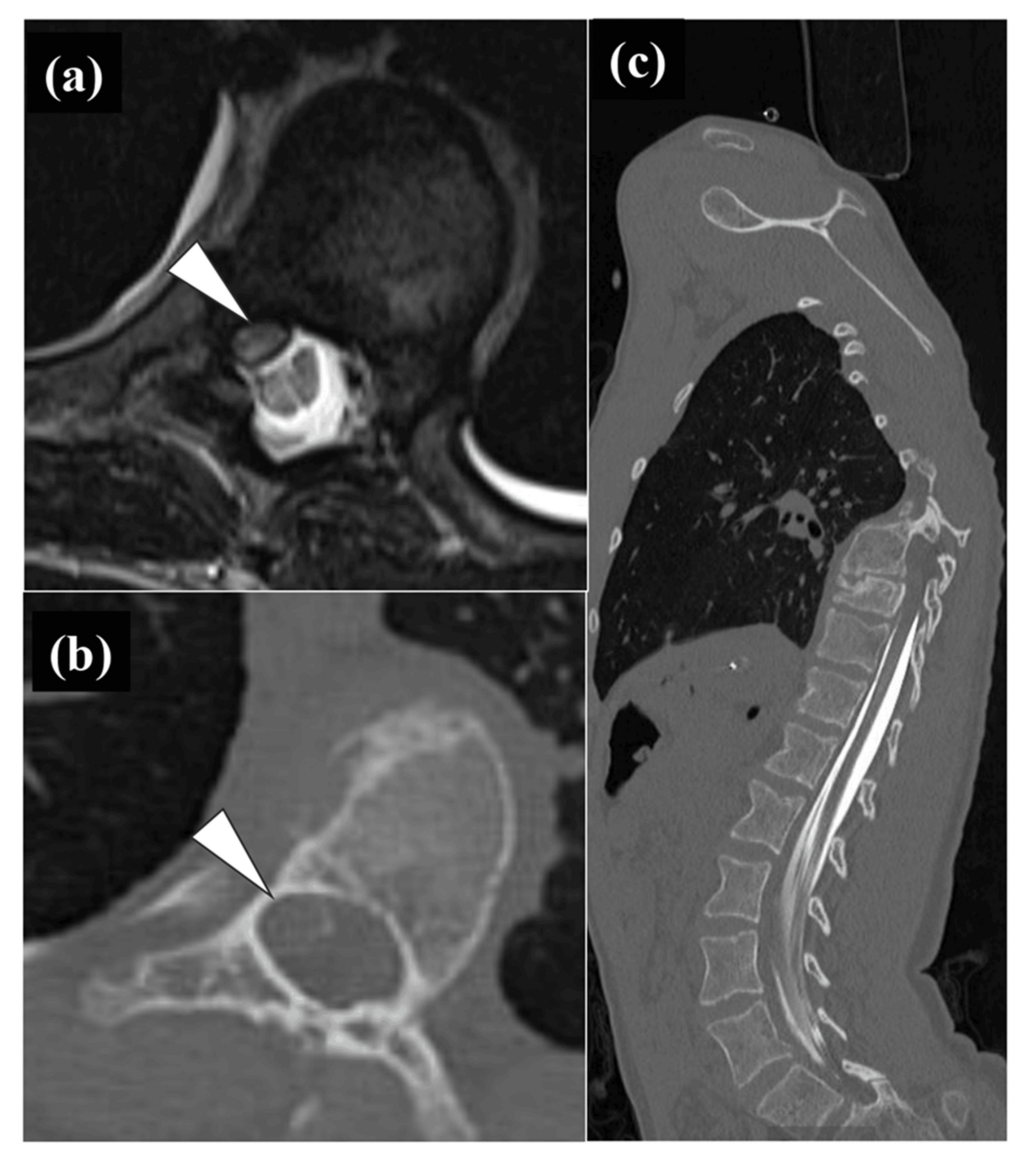
Preoperative magnetic resonance (MR) and computed tomographic myelograhic (CTM) image Axial MR image also showed the deviated and compressed spinal cord (a). CTM image showed that the spinal cord was deviated to the concave side and was compressed from the right pedicle and posterior wall of the vertebral body at T10 level in axial plane (b), and the contrast medium injected into the dural sac from L4/5 level did not flow cephalad from T10 in sagittal plane (c).The white triangles in (a) and (b) indicated the dural sac and spinal cord.

**Figure 3:**
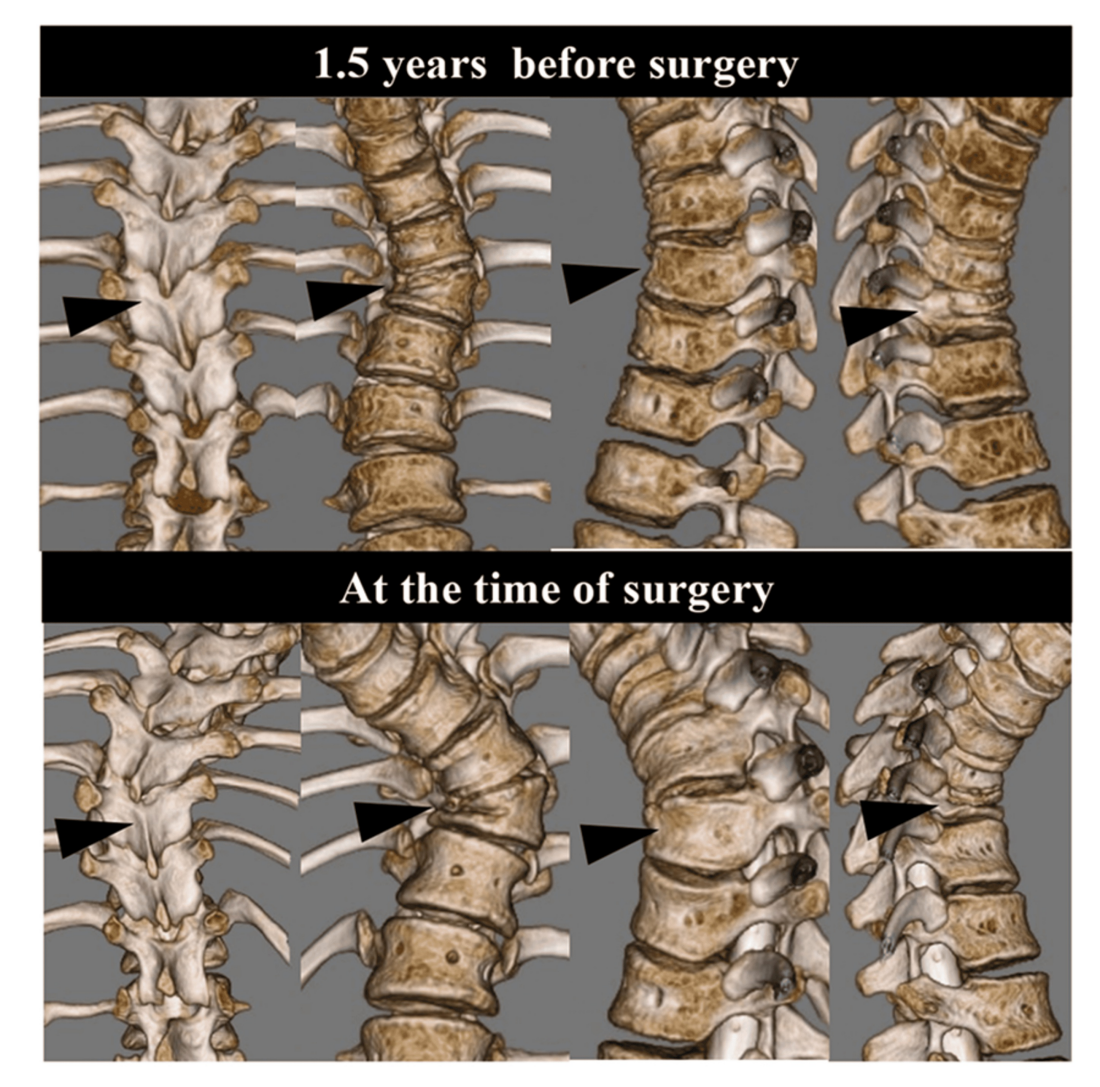
Three-dimensional computed tomographic (3DCT) images 1.5 years before and at the time of surgery The 3DCT images in the upper column were obtained 1.5 years before surgery, while the 3DCT images in the lower column were taken at the time of surgery. Both images were viewed from posterior, anterior, left, and right, respectively, from the left. The T10-wedged vertebra, indicated by the black triangle, exhibited a notable degree of deformation and remarkable progression of kyphoscoliosis during this interval.

The case presented here was a 12-year-old male. At the age of two years, Sotos syndrome was suspected due to congenital heart disease, psychomotor developmental delay, and characteristic facial features. Chromosome analysis with fluorescence in situ hybridization revealed a 5q35 deletion, which confirmed the diagnosis of Sotos syndrome. At the age of three years, scoliosis was noted by his pediatrician and he was referred to the pediatric orthopedic surgery outpatient clinic. Thereafter, the scoliosis did not progress significantly and he was followed up by a pediatric orthopedic surgeon, including regular radiographic imaging. A year and a half ago, he was referred to the author's scoliosis outpatient clinic because his scoliosis curve became 35 degrees. Because of the steep curve, computed tomography (CT) scan was added. The three-dimensional CT images revealed a concomitant wedged vertebra. Since the degree of the curve was not large, the patient continued to be followed without bracing therapy by radiographs in the author's outpatient clinic. At the age of 12, the Cobb angle reached 60 degrees, and the author suggested to his parents that surgery might be considered in the near future. Until that time, there was no specific mention of the appearance of neurological symptoms. Two months later, he and his mother suddenly came to the outpatient clinic because his neurological symptoms had become apparent. He began to have gait disturbance and urinary disturbance (Video 1). Deep tendon reflexes in the lower extremities were bilaterally hyperactive, and ankle clonus was bilaterally positive. However, sensory impairment and the grade of manual muscle test could not be assessed exactly because of his mental retardation and the disease characteristic of a congenital weak muscle tone. Standing full-spine radiographs showed a scoliotic major curve in the thoracolumbar transition region and kyphoscoliosis with T10 as the apex (Figure 1a). The Cobb angle of the coronal major curve was 60 degrees and that of the sagittal local kyphosis was 60 degrees (Figure 1a,b). Both coronal and sagittal views showed rapid worsening of kyphoscoliosis over the course of 1.5 years, and the triradiate cartilage (TRC) was also closed during this period (Figure 1a-d). Magnetic resonance imaging (MRI) showed that the spinal cord was deviated to the concave side and was compressed from the right pedicle and posterior wall of the vertebral body at T10 level (Figure 2a). Three-dimensional images of CT images showed a wedged vertebra at T10 (Figures 2b, 3). It was noteworthy that the contrast medium injected into the dural sac from L4/5 level did not flow cephalad from T10 in the CT image of myelography (Figure 2c). Based on these findings, myelopathy due to exacerbation of kyphoscoliosis was diagnosed, and it was decided to perform direct decompression and posterior corrective fixation, including T10 pediculectomy and partial vertebral resection.

**Video 1:** Ambulatory status before surgery The mother holds up a smart phone, but he could only get it by crawling on all fours; he had difficulty walking. Also of note were the macrocephaly, characteristic facial features, and long limbs that were characteristic of Sotos syndrome.

**Figure 4:**
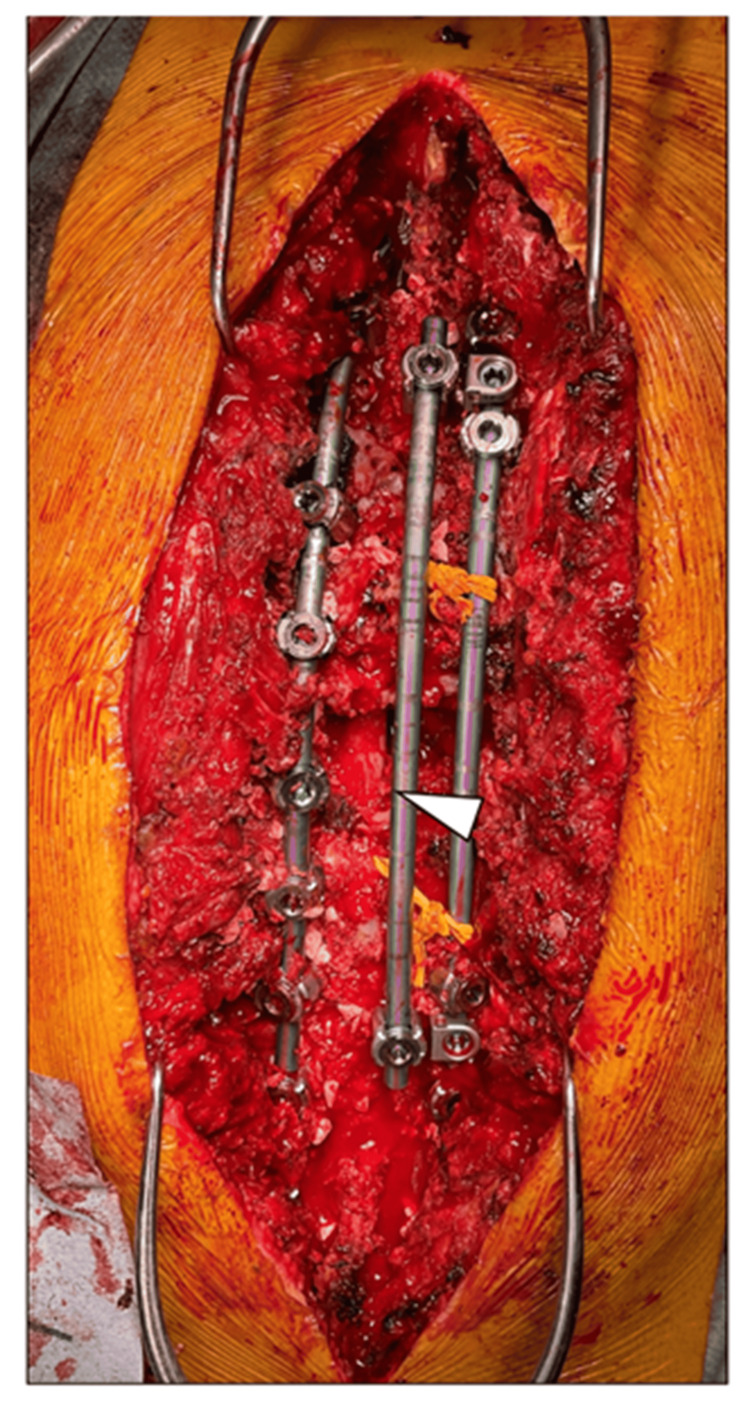
Intraoperative photograph The white triangle indicates dural sac.

Surgery was performed under general anesthesia in the prone position on a Jackson spinal table. He received 50 mg/kg of diluted tranexamic acid in normal saline solution to a volume of 1 mL/kg infused over 15 minutes, followed by a maintenance infusion of 10 mg/kg/h of 100 mg/kg concentration (0.1 mL/kg/h) according to the previous report [5], and intraoperative monitoring using the transcranial electric stimulation-motor-evoked potentials were recorded. However, the waveform was very unstable, and amplitude was low and could not be recorded adequately. A median longitudinal incision was made and expanded to the transverse process level. Polyaxial pedicle screws were placed segmentally using a navigation system (Stealth Station S7, Medtronic Sofamor Danek, Memphis, TN) with preoperative CT images. Titanium (Ti) rod 6.0 mm in diameter was temporally placed from T9 to T11 on the left. Laminectomy from T9 to T10 was performed to expose the dural sac, followed by facet resection of the bilateral T9/10 and 10/11, leaving only the right T10 pedicle. After the right T10 rib was resected from the rib head about 3 cm from the vertebral body, the lateral side of the T10 vertebral body was resected while preserving the inner wall of the pedicle as much as possible to prevent the spinal cord from shifting outward and interfering with the operation. As a precaution, a penfield was inserted at the inner edge of the thinning pedicle to protect the dural sac. After ligating the right T10 nerve root, the central end was gently raised contralaterally with No. 1 silk thread, and the vertebral body was resected laterally and anteriorly. After excavating the T10 vertebral body laterally about 1 cm toward the midline, the pedicle was resected with a punch, and the dural sac was further shifted to the right side. While protecting the dural sac with a penfield, the posterior wall of the vertebral body was additionally resected with a punch, and sufficient decompression was performed. The left T10 nerve root was found to be taut, which could have shifted further to the right side if transected, so it was preserved instead of transection [6]. The remaining facet joints of T5/6-T13/L1 were released by resection of the inferior articular process and also underwent indirect decompression by corrective fixation with 6.0-mm diameter Ti rod. After correction, the dural sac was found to have shifted to the midline (Figure 4). Sublaminar wires were placed in the T8 and T11 laminae and additional rod were placed to enhance fixation. Morselized local bone was grafted on the laminae. One week later, anterior bone graft at T9/10- and 10/11-disc levels was performed, following thoracotomy with resection of the T8 rib.

**Figure 5:**
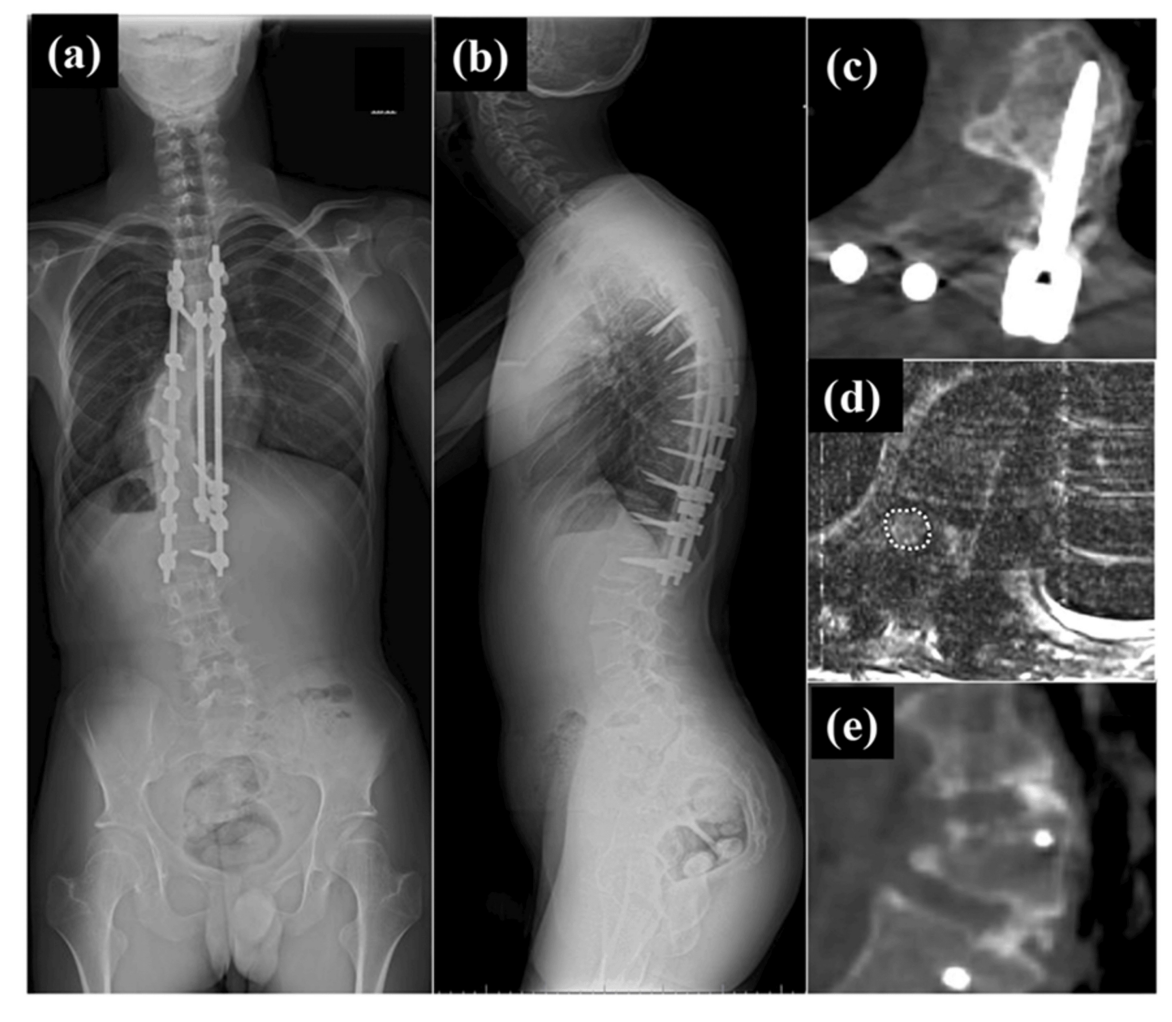
Postoperative coronal and sagittal standing full-spine radiograph, computed tomographic (CT) and magnetic resonance (MR) images At 1.5 years after surgery, coronal standing full-spine radiograph showed the Cobb angle of the scoliotic major curve from T8 to T11 was 40 degrees (a), and sagittal standing full spine radiograph showed local sharp kyphosis, whose Cobb angle was 45 degrees, became less outstanding and thoracic kyphosis (T5-12) was 50 degrees (b). Axial CT image at one year after surgery showed that the right T10 pedicle and the part of the posterior and lateral walls of the T10 vertebral body was resected. (c). Axial MR image at six months after surgery showed the spinal cord (area enclosed by white dashed line), which had been as flattened preoperatively, was depicted as an oval (d). Coronal CT image at one year after surgery showed bone union was obtained in T9/10 and 10/11 levels performed with anterior bone grafting.

After the surgery, the standing full-spine radiograph showed scoliosis improved to 40 degrees and local kyphosis improved to 45 degrees, and spinal cord compression was found to be relieved in CT image and MRI (Figure 5a-d). One-year postoperative CT images also showed bone union (Figure 5e). His gait disturbance had improved (Video 2), and he no longer had urinary incontinence at 1.5 years after surgery.

**Video 2:** Ambulatory status after surgery The video was taken about three months after surgery. He was able to walk and retrieve a smartphone held up by his mother.

## Discussion

Few studies have reported the outcome of a large number of cases of Sotos syndrome, with the study by Corrado et al. being the most numerous [1]. They diagnosed 42 cases of Sotos syndrome, of which 8 (19%) had scoliosis of more than 10 degrees or kyphosis of more than 40 degrees. Because the progression of scoliosis was rapid by four years of age, which corresponds to the characteristic period of overgrowth in Sotos syndrome, there are cases in which scoliosis curves are large and require therapeutic intervention from the initial examination, even at a young age. Various types of scoliosis curves have been reported, including thoracic or lumbar single curves, thoracic and lumbar double curves and triple curves [1,7-9]. There are reports of bracing therapy to delay the timing of surgery, but it is difficult to control curve progression with orthotics [1,2]. Corrado et al. stated that bracing therapy was performed to delay the timing of surgery, but ultimately surgery was performed in seven cases. Recently, there has been a report on the results of growth-friendly surgery. In that report, the incidence of complications, including implant-related issue, in growth-friendly surgery for Sotos syndrome appeared to be similar to patients with syndromic or neuromuscular causes of early onset scoliosis (EOS) [7]. Although proximal junctional kyphosis (PJK) and proximal hook dislodgement are possible complications in definitive fixation as well as growth-friendly surgery, they are not specific to this syndrome, but the hyperextension of the joints and hypotonia that characterize this syndrome may also play a role. Therefore, rather than employing a short fusion as is typically done in idiopathic scoliosis, a longer fusion is recommended to minimize the risk of such complications at the time of the definitive fixation [1]. In the present case, bracing therapy was not actively considered because of the coexistence of wedged vertebra. The TRC was closed when the scoliosis curve reached 60 degrees, and the timing of the definitive fixation was to be discussed with the parents, as we thought that the final fixation surgery was more indicated than the growth-friendly surgery. With regard to the extent of fixation, it might have been preferable to place lower instrumented vertebra at L3, the stable vertebra, or lower. However, given that the primary objective of the surgery was to improve paraparesis, indirect decompression by correcting the thoracic major curve, in addition to direct decompression, was considered to be the most appropriate approach. If the lumbar curve were to increase postoperatively, additional surgery could be considered. One and a half years after surgery, there was no evidence of implant failure, or progression of lumbar curve and PJK. In addition, it was confirmed by CT image that bone union had been achieved at the surgical site.

McMaster et al. found that the average kyphosis at the onset of paralysis was 111 degrees but one patient who had a posterolateral quadrant vertebra at the ninth thoracic level had only a 60° among 112 cases of congenital scoliosis and kyphoscoliosis [8]. In that case, the patient was diagnosed with a posterolateral quadrant hemivertebra case, in which typically presents with rapid curve progression. However, the present case was diagnosed with a wedged vertebra, which is considered to have a relatively slow curve progression, and in that respect, it was very rare because the curve progressed rapidly and paraplegia occurred despite a less severe deformity with local kyphosis of about 60 degrees. 

It has been reported that most cases of paraplegia are due to formation failure in the thoracolumbar transition area [8]. It was speculated that if there is a formational failure, especially quadrant hemivertebra, that causes local kyphosis as well as scoliosis in the thoracolumbar transition area, the compensatory lumbar lordosis changes are likely to result in sharp angularity of the spinal cord. Furthermore, diseases based on muscle hypotonia, such as Sotos syndrome, often present with kyphoscoliosis and are more likely to be loaded anteriorly on the vertebral body than usual. Therefore, according to the Hueter-Volkmann law, the anterior concave side of the vertebrae is suppressed while the posterior convex side tends to overgrow, and the deformity of the wedged vertebrae is expected to progress more significantly than expected in the natural course. In fact, the wedge changes rapidly progressed on the image over the course of 1.5 years. As a result, it was speculated that a high degree of sharp angular deformity occurred, resulting in myelopathy. In reviewing previous reports of Sotos syndrome [1-2,7,9], there were no cases of spinal disorder or alterations of spinal cord in MRI during the course of treatment or follow-up, and it was considered that spinal cord disorder in the present case was solely the result of a combination of the two congenital syndromes: congenital vertebral anomaly and Sotos syndrome.

In cases of kyphosis and kyphoscoliosis with congenital formation failure, McMaster et al. recommended posterior fixation as early as five years of age, before the local kyphosis exceeds 40° to 50° [8]. In cases of kyphosis that exceeds 50° even in the supine position, anterior-posterior combined corrective fixation, including anterior release and bone grafting, is recommended. In cases where a neurological disorder is present, if the disorder is mild enough to allow the patient to walk unassisted, indirect decompression with correction should be performed, but if the disorder is severe enough to make walking unassisted impossible, direct decompression including partial vertebral body resection and pediculectomy, although there are risks associated with the surgical technique. In consideration of the aforementioned factors, the timing of the surgical intervention in this case was regrettably delayed. However, it was considered that the surgical technique employed was appropriate.

## Conclusions

Two congenital diseases, Sotos syndrome and congenital vertebral anomaly, such as the present case, require careful follow-up or early surgical intervention, especially during the growth-spurt period, to avoid missing the good timing for surgery because of the possibility of rapid deterioration of kyphoscoliosis and subsequent spinal cord disorders. In cases of severe spinal cord disorders resulting in the inability of the patient to walk unassisted, not only indirect but also direct decompression is recommended despite the high technical demands.
